# How corrective messages persuade in AI era: message sidedness, source attribution, and psychological pathways in health misinformation correction

**DOI:** 10.3389/fpsyg.2026.1800821

**Published:** 2026-08-31

**Authors:** Jiaxiang Xu, Jiayuan Yu, Wanhui April Zhou, Yinqiao Zhao, Jingyuan Shi

**Affiliations:** 1 School of Communication, Hong Kong Baptist University, Kowloon, Hong Kong SAR, China; 2 Department of Interactive Media, Hong Kong Baptist University, Kowloon, Hong Kong SAR, China

**Keywords:** AI in health communication, flu vaccine, health misinformation correction, message sidedness, persuasion process, source credibility

## Abstract

**Introduction:**

In contemporary AI-mediated digital environments, corrective health information is encountered through diverse sources and message formats, raising questions about how message design and source attribution shape belief endorsement. Drawing on persuasion theory, this study examines the effects of corrective message sidedness and source attribution on endorsement of a corrected health claim.

**Methods:**

A 2 × 3 factorial experiment was conducted with 543 participants. Corrective message sidedness was manipulated as one-sided or two-sided, while source attribution identified the correction as originating from an LLM chatbot, a government health authority, or an unspecified source. The correction addressed a health claim concerning influenza vaccination during pregnancy. Factorial analysis of variance was used to test the main and interaction effects of message sidedness and source cues. Mediation analyses were conducted to examine the roles of perceived persuasiveness and perceived source credibility.

**Results:**

Significant main effects were found for both message sidedness and source cues. Two-sided corrective messages generated higher belief endorsement than one-sided messages, while corrections attributed to a government health authority generated higher belief endorsement than those attributed to an LLM chatbot or an unspecified source. The interaction between message sidedness and source cues was not significant. Mediation analyses further showed that perceived persuasiveness mediated the effect of message sidedness on belief endorsement, whereas perceived source credibility mediated the effect of source attribution.

**Discussion:**

Corrective health messages influence belief endorsement not only through the information they provide but also through distinct psychological processes associated with argument evaluation and credibility inference. The findings advance understanding of health misinformation correction in AI-mediated environments and provide practical guidance for designing more effective corrective messages in digital health communication.

## 1 Introduction

Correcting health misinformation does not guarantee that individuals will accept the correction. Even when false claims are explicitly rebutted, people may remain unconvinced, selectively interpret evidence, or continue to rely on prior beliefs (Ecker et al., 2022; Walter et al., 2020). This gap between exposure to accurate information and genuine belief revision represents one of the central psychological challenges in contemporary health communication. The problem has grown increasingly consequential as individuals navigate complex digital environments filled with competing narratives about medical risks, treatments, and preventive behaviors. This challenge is particularly salient in health misinformation correction, where the goal is not merely to present accurate information but to help recipients reconsider contested health claims in environments where false information can circulate widely. Studies of health misinformation correction on social media show that corrections can reduce misperceptions, but their effectiveness depends on who provides the correction, how the correction is delivered, and whether audiences perceive the corrective information as credible (Bode and Vraga, 2018; Vraga and Bode, 2017). More broadly, work on health misinformation emphasizes that effective correction requires attention not only to factual accuracy, but also to source credibility, message design, and the platform environments in which audiences encounter competing claims (Chou et al., 2018; Vraga and Bode, 2020). This makes health misinformation correction a useful context for examining how message structure and source attribution jointly shape acceptance of corrective information.

More recently, the rise of artificial intelligence (AI), particularly large language models (LLMs) that provide immediate and seemingly authoritative responses to health inquiries, has further transformed how corrective information is delivered and evaluated (Yun and Bickmore, 2025). While these technologies expand access to health knowledge, they also heighten the stakes of persuasive communication, because when misinformation is corrected, the structural features and source attribution of corrections may influence whether individuals accept the information or continue to rely on false claims. Understanding what makes corrective messages psychologically compelling is therefore not merely a theoretical concern but a pressing public health priority. Additionally, in traditional correction contexts, source cues often derive from identifiable actors such as health authorities, experts, or media organizations. LLM chatbots complicate this assumption because they may signal technical competence and informational efficiency while lacking stable institutional accountability and intentional agency. In this context, LLM chatbots raise a theoretical question for corrective persuasion research: whether source-based credibility mechanisms developed around human and institutional sources can be generalized to algorithmic source attribution.

Research on misinformation correction has traditionally emphasized the importance of factual rebuttals, evidence quality and source expertise, demonstrating that perceived credibility of source plays a key role in whether corrective information is accepted (Walter et al., 2020). Yet correction effectiveness cannot be fully explained by content accuracy or source authority alone (Lewandowsky et al., 2012). Recipients may also evaluate whether the corrective argument appears persuasive and whether the attributed source appears credible. Message structure and source attribution are therefore central to corrective persuasion because they may activate different evaluative processes through which recipients judge whether a correction should be accepted. One structural feature with longstanding relevance in persuasion research is message sidedness (O’Keefe, 1999). One-sided messages present only arguments supporting a focal claim, whereas two-sided messages introduce opposing viewpoints before addressing them. Importantly, scholars distinguish between two forms of two-sided messaging: non-refutational two-sided messages, which acknowledge counterarguments without directly challenging them, and refutational two-sided messages, which explicitly rebut opposing claims after presenting them (Allen, 1991; O’Keefe, 1999).

Classic persuasion theories offer insight into why such structural differences matter. When individuals evaluate contested claims, they often rely on cues that help them determine whether a message reflects thorough consideration or selective presentation. A balanced corrective message can function as a diagnostic signal that the communicator has engaged with alternative perspectives, potentially enhancing perceptions of argument strength (Wallace et al., 2024). Early persuasion research suggested that two-sided messages may sometimes be less effective than one-sided appeals because introducing opposing arguments can generate doubt or increase cognitive burden (Hovland et al., 1949). However, more recent empirical evidence suggested that refutational two-sided messages can be useful in misinformation context, especially when they acknowledge the audience’s existing belief before directly rebutting it. For example, Xu and Petty (2025) found that two-sided correction messages were especially effective among individuals with stronger misinformation beliefs, partly because such messages increased appreciation of the source’s acknowledgment and credibility. Health-related studies have similarly examined sidedness in corrections about e-cigarettes and HPV vaccination, suggesting that the effectiveness of one-sided versus two-sided correction may depend on prior experience, topic involvement, and the specific correction format (Wang and Huang, 2021; Xiao and Su, 2021). Despite this advantage, the effectiveness of two-sided refutation remains context dependent. For example, in advertising settings where audiences often engage in low-effort processing, raising counterarguments may inadvertently highlight product weaknesses and reduce persuasive impact (Crowley and Hoyer, 1994). By contrast, in contested informational environments such as misinformation correction, refutational fact-checks can directly address the false claim while presenting corrective information, a structure that may help recipients distinguish the misinformation from the correction (Ecker et al., 2020).

These findings suggest that the persuasive value of message sidedness cannot be understood solely by comparing one-sided and two-sided formats. Rather, the effect of sidedness depends on how recipients interpret the message in relation to the issue, their prior beliefs, and the credibility of the communication context (Xu and Petty, 2024). In health misinformation correction, this interpretive process is especially important because audiences may already have encountered competing claims and may evaluate whether the correction appears responsive to those claims. Thus, the key theoretical question is not only whether two-sided corrections outperform one-sided corrections, but also why such a structure may make a correction more acceptable. Accordingly, two-sided corrections are not inherently more effective than one-sided corrections; rather, they may influence belief endorsement by first increasing perceived persuasiveness. This mechanism helps explain why empirical findings on message sidedness have been mixed, as the persuasive impact of message structure depends on the extent to which recipients attend to and process the arguments presented. This distinction becomes theoretically consequential in AI-mediated correction environments. If AI-attributed corrections lack the inherited institutional legitimacy of conventional health authorities, message design may represent an alternative route through which recipients evaluate the correction. However, whether this message-structure pathway operates differently across AI and institutional source attributions remains an empirical question. In that sense, examining message sidedness in an AI-era context does more than reproduce an established persuasion effect in a new setting; it helps clarify whether structural features of correction remain influential even when source-based credibility is less securely anchored.

In addition to message structure, recipients often rely on source attribution to evaluate the trustworthiness of corrective information, particularly in environments characterized by epistemic uncertainty. Source cues function as heuristic signals that help individuals infer whether information is credible, especially when they lack the expertise to independently verify its accuracy (Cacciatore, 2021). Traditionally, institutional sources such as government health agencies have been perceived as authoritative because they are associated with expertise, professional responsibility, and public accountability (Holroyd et al., 2020). These assumptions, however, become more complex when corrective information is attributed to LLM chatbots. As LLM-based chatbots become increasingly visible in online health information seeking, they are beginning to function alongside traditional sources such as search engines and health websites (Yun and Bickmore, 2025). Their conversational, immediate, and seemingly personalized responses may enhance perceived usefulness and accessibility, but evaluating LLM-based health information involves more than judging informational quality alone. Recent frameworks for human evaluation of healthcare LLMs emphasize dimensions such as safety, expression style, and trust and confidence (Tam et al., 2024). Evidence from AI-assisted misinformation correction similarly cautions against assuming that AI cues automatically enhance correction effectiveness. In a preregistered two-wave experiment on raw milk disinformation, In a preregistered two-wave experiment on raw milk disinformation, Tang et al. (2025) manipulated source expertise, AI attribution, and correction placement, finding that debunking corrections reduced misperceptions and behavioral intentions for at least 1 week, whereas AI attribution generally showed limited effects on correction effectiveness. However, their findings also suggest that the influence of AI cues may depend on contextual factors, such as source expertise and correction placement: for example, AI attribution reduced the effectiveness of corrections when an expert organization’s information was presented proactively through prebunking, but not when AI-supported corrections directly addressed misinformation claims. Another related source-disclosure research also shows that disclosing AI authorship can shape evaluations of AI-generated health prevention messages. In a two-part study on vaping prevention messages, Lim and Schmälzle (2024) found that source disclosure affected message evaluations and produced a slight overall bias against AI-generated messages, although it did not consistently change message rankings. Taken together, this body of work suggests that AI attribution is theoretically ambiguous in health misinformation correction: it may signal technical competence and informational efficiency, while also raising skepticism about whether the source is medically responsible, accountable, and trustworthy, especially in domains where people are already cautious about medical AI (Longoni et al., 2019).

Unlike conventional institutional sources, LLM chatbots do not derive credibility from formal accountability, professional responsibility, or stable public authority. Instead, they are encountered through technologically mediated interfaces that present information as machine-generated or machine-delivered. This distinction is important because LLM chatbots differ from health authorities in the basis on which credibility is inferred. A government health authority possesses an identifiable institutional identity, professional mandate, and public accountability. By contrast, an LLM chatbot may signal technical competence, computational capacity, and informational efficiency, but it does not possess intentional agency or stable institutional responsibility in the same way as a public health organization. Therefore, LLM chatbot attribution should be understood as a technological cue that may activate credibility-related inferences while also raising uncertainty about accountability, expertise, and responsibility.

Importantly, source attribution cues may play a limited role to influence belief endorsement solely by virtue of the label itself. Rather, they shape belief updating in part by informing recipients’ credibility-related inferences about the corrective message, while potentially coexisting with other evaluative processes (Metzger et al., 2010). From this perspective, source attribution may function as an entry point into persuasion, orienting recipients toward the message and shaping their willingness to accept or scrutinize its content. Accordingly, perceived source credibility refers to recipients’ subjective evaluation of the attributed source rather than an objective property of the source itself, may function as a mediating role between source attribution and belief endorsement. Even when the attributed source is algorithmic, recipients may still judge whether it appears knowledgeable, trustworthy, and reliable. From this perspective, source-attribution cues may influence post-exposure endorsement not merely through the presence of a label, but by shaping credibility-related inferences about the correction provider. In contrast to message sidedness, which is expected to operate by shaping evaluations of argument strength and balance, source attribution is expected to operate primarily by shaping perceived source credibility (Holroyd et al., 2020).

Beyond their independent effects, message sidedness and source attribution may also interact because source labels can shape how recipients interpret message structure. From the perspective of persuasion knowledge, individuals may evaluate corrective messages differently depending on whether the source appears institutionally accountable or algorithmically generated (Friestad and Wright, 1994). A correction attributed to a government health authority may benefit from preexisting institutional credibility, making recipients more willing to accept even a relatively direct one-sided rebuttal. By contrast, an AI-attributed correction may trigger greater uncertainty about expertise, accountability, or algorithmic reliability (Dietvorst et al., 2015; Longoni et al., 2019). Under such conditions, a refutational two-sided structure may become especially consequential because acknowledging and rebutting opposing claims can signal transparency, balance, and careful reasoning. At the same time, social responses to computers and algorithmic trust research suggest that recipients may not interpret an LLM chatbot’s balanced reasoning in the same way they interpret a health authority’s balanced reasoning (Nass and Moon, 2000; Sundar, 2008). Therefore, rather than predicting a specific direction, we examine whether message sidedness and source attribution interact in shaping post-exposure endorsement of the corrected claim.

Taken together, the present study contributes to corrective persuasion research in three ways. First, it offers a contextual generalization of message-sidedness research by testing whether refutational two-sided corrections remain more persuasive in an AI-mediated health misinformation context. Second, it qualifies source credibility models by comparing a traditional institutional source with an AI algorithmic source cue and an unspecified source, thereby examining whether AI attribution carries the same credibility-based persuasive function as health authority attribution. Third, it separates two evaluative pathways through which corrections may be accepted: a message-structure pathway, in which sidedness shapes perceived persuasiveness, and a source-attribution pathway, in which source labels shape perceived source credibility. This framework clarifies how established persuasion mechanisms operate, and where they may be limited, when health corrections are attributed to LLM chatbots. We thus proposed the following hypothesis and questions:

*H1*: The two-sided corrective message will produce higher belief endorsement toward the message than the one-sided corrective message.

*RQ1*: How do source attribution influence belief endorsement? Specifically, how do corrections attributed to LLM Chatbot and an unspecified online source compare with those attributed to a health authority?

*RQ2*: Does message sidedness interact with source attribution to influence belief endorsement?

*RQ3*: Does perceived persuasiveness mediate the relationship between message sidedness (one-sided vs. two-sided corrections) and belief endorsement in a health misinformation correction context?

*RQ4*: Does perceived source credibility mediate the relationship between source attribution (LLM chatbot, health authority, or unspecified source) and belief endorsement following exposure to corrective health information?

## 2 Materials and methods

### 2.1 Study design

This study employed a 2 × 3 factorial experimental design manipulating message sidedness (one-sided vs. two-sided corrective information) and source attribution (artificial intelligence, health authority, and unspecified online source), resulting in six experimental conditions. Participants were randomly assigned to one of the six conditions.

### 2.2 Participants

Data were collected through Credamo, a large Chinese online research platform. Eligible participants were adults aged 18 years and older. A total of 543 valid responses were retained after excluding cases that failed attention checks or exhibited abnormally short completion times. The final sample included 56.7% females (*N* = 308) and 43.3% males (*N* = 235), with ages ranging from 18 to 70 years (*M* = 31.54, SD = 8.16).

### 2.3 Materials and procedure

Participants were randomly assigned to one of six experimental conditions. Each participant was first exposed to a piece of health misinformation stating that “influenza vaccination is not recommended for pregnant women due to potential risks to both the mother and the fetus,” the misinformation was followed by a corrective message that varied in sidedness. This topic was selected because it represents a consequential health misinformation context involving preventive behavior, risk perception, and public health recommendation, making it suitable for examining corrective message effects.

In the one-sided message condition, the corrective message presented only supporting evidence for vaccination safety. In the two-sided message condition, the message first acknowledged common concerns about vaccination risks before refuting them with evidence-based information. Source attribution was manipulated by presenting the corrective message as originating from one of three sources: LLMs Chatbot (DeepSeek, a famous Chinese large language model), a government health authority (National Health Commission of China), or unspecified online source. Source information was conveyed through both textual labels (e.g., “Information source: Artificial Intelligence”) and corresponding visual logos to enhance manipulation salience.

After reading the materials, participants completed measures assessing belief endorsement, perceived persuasiveness, and source credibility. Participants also reported demographic information including gender, age, education level, and income.

### 2.4 Manipulation checks

Manipulation checks were conducted using matrix-style items assessing participants’ recall of the message source and the structure of the corrective content. Over 92% of participants correctly identified the source attribution, and more than 88% accurately recognized the message structure, indicating successful manipulation of both experimental factors.

### 2.5 Measurement

Belief endorsement was assessed using a single-item measure. After reading the misinformation correction, participants were presented with the corrected statement “Pregnant women can safely receive the influenza vaccine” and asked to indicate the extent to which they believed the statement was correct on a 7-point scale (1 = totally wrong, 7 = totally right). This single-item measure was used to directly assess endorsement of the focal corrected factual claim, an approach commonly used in misinformation correction research when the outcome concerns belief in a specific claim. Perceived source credibility was measured using a 7-point Likert scale adapted from Sussman and Siegal (2003). Items assessed participants’ perceptions of the source’s expertise and trustworthiness. Sample question: “To what extent is the source who wrote this message an expert on the message topic?” (*α* = 0.89). Perceived persuasiveness was measured using four items adapted from Zhang et al. (2014), rated on a 7-point Likert scale (1 = strongly disagree, 7 = strongly agree). Sample items included “The arguments in this message were convincing” and “The arguments in this message were persuasive” (*α* = 0.93).

### 2.6 Statistics

We analyzed the primary outcome, belief endorsement, using a 2 × 3 factorial ANOVA to examine the main and interaction effects of message sidedness (one-sided vs. two-sided) and source cues (AI, health authority, plain text). To further investigate the psychological mechanisms underlying these effects, we conducted two parallel mediation analyses using Hayes’ PROCESS model. Because two mediation models were estimated, we applied a Bonferroni-adjusted significance threshold (α = 0.025) to reduce the risk of Type I error. All indirect effects were estimated using 5,000 bootstrap samples with bias-corrected 95% confidence intervals.

## 3 Results

Table 1 presents the descriptive statistics, Pearson correlations and Cronbach’s α coefficient among the study variables. The multi-item measures demonstrated satisfactory internal consistency. Belief endorsement was positively associated with the two proposed mediators, perceived source credibility and perceived persuasiveness.

**Table 1:** Means (M), standard deviations (SD) and intercorrelations between all variables.

Variable	*M*	*SD*	1	2	3	4	5	6	7
1. Belief endorsement	5.503	1.237							
2. Perceived source credibility	5.142	1.219	0.647***						
3. Perceived persuasiveness	5.225	1.326	0.751***	0.849***					
4. Age	31.54	8.157	0.07	0.121**	0.073				
5. Gender			0.036	0.057	0.009	0.138**			
6. Education			0.124**	0.011	0.051	−0.061	−0.111**		
7. Income			0.165***	0.086*	0.143***	0.207***	−0.04	0.318***	

*p < 0.05, **p < 0.01, ***p < 0.001.

### 3.1 Effects of message sidedness and source attribution on belief endorsement

Prior to the main analysis, we examined the homogeneity of variance assumption. Levene’s test suggested some heterogeneity of variance, *F*(5, 537) = 2.911, *p* = 0.013; however, prior methodological work indicates that ANOVA is relatively robust to moderate violations of this assumption, particularly when the sample size is reasonably large and cell sizes are approximately balanced (Glass et al., 1972; Wang et al., 2017). Therefore, the factorial ANOVA was retained as the primary analytic approach and interpreted with caution.

A two-way ANOVA was conducted to examine the effects of message sidedness (one-sided vs. two-sided messages) and source attribution (LLM chatbot, health authority, unspecified) on belief endorsement toward the corrective message. The analysis revealed a significant main effect of message sidedness, *F*(1, 537) = 8.998, *p* = 0.003, η^2^ = 0.016. Although statistically significant, the effect size was small, indicating a modest difference in endorsement between the two message conditions. Post-hoc analysis shown that participants exposed to two-sided corrections reported higher endorsement (*M* = 5.65, SD = 1.23) than those exposed to one-sided corrections (*M* = 5.34, SD = 1.28), M_diff_ = 0.314, *p* = 0.003.

A significant main effect of source attribution was also emerged, *F*(2, 537) = 5.636, *p* = 0.004, η^2^ = 0.020. This effect was also small in magnitude, suggesting that source attribution produced a statistically significant but modest difference in post-exposure endorsement. *Post hoc* comparisons indicated that corrections attributed to the health authority produced significantly higher belief endorsement than those labelled as LLM chatbot (M_diff_ = 0.414, p = 0.004) and unspecified (M_diff_ = 0.312, *p* = 0.042). No significant difference was observed between LLM Chatbot and unspecified corrections (*p* = 0.706). These findings suggest that traditional institutional attribution conferred a modest endorsement advantage over LLM chatbot attribution and unspecified attribution in this health misinformation correction context (see Tables 2, 3).

**Table 2:** Effects of message sidedness and source attribution on belief endorsement.

Effect	*F*(df1, df2)	η^2^	*p*	95%CI	
				Lower	Upper
Message sidedness	*F*(1, 537) = 8.998	0.016	0.003**	0.002	0.043
Source attribution	*F*(2, 537) = 5.636	0.020	0.004**	0.002	0.047
Message sidedness × source attribution	*F*(2, 537) = 0.764	0.003	0.466	0.000	0.015

*p < 0.05, **p < 0.01, ***p < 0.001. Values represent results from a 2 × 3 between-subjects ANOVA. Message sidedness (one-sided vs. two-sided) and source attribution (LLM chatbot, health authority, unspecified) were entered as fixed factors.

**Table 3:** Means and standard deviations of belief endorsement by message sidedness and source attribution.

Source	One-sided M (SD)	Two-sided M (SD)
LLM Chatbot	5.091 (1.467)	5.557 (1.154)
Health authority	5.663 (1.096)	5.813 (1.098)
Unspecified	5.262 (1.131)	5.589 (1.317)

**Figure 1:**
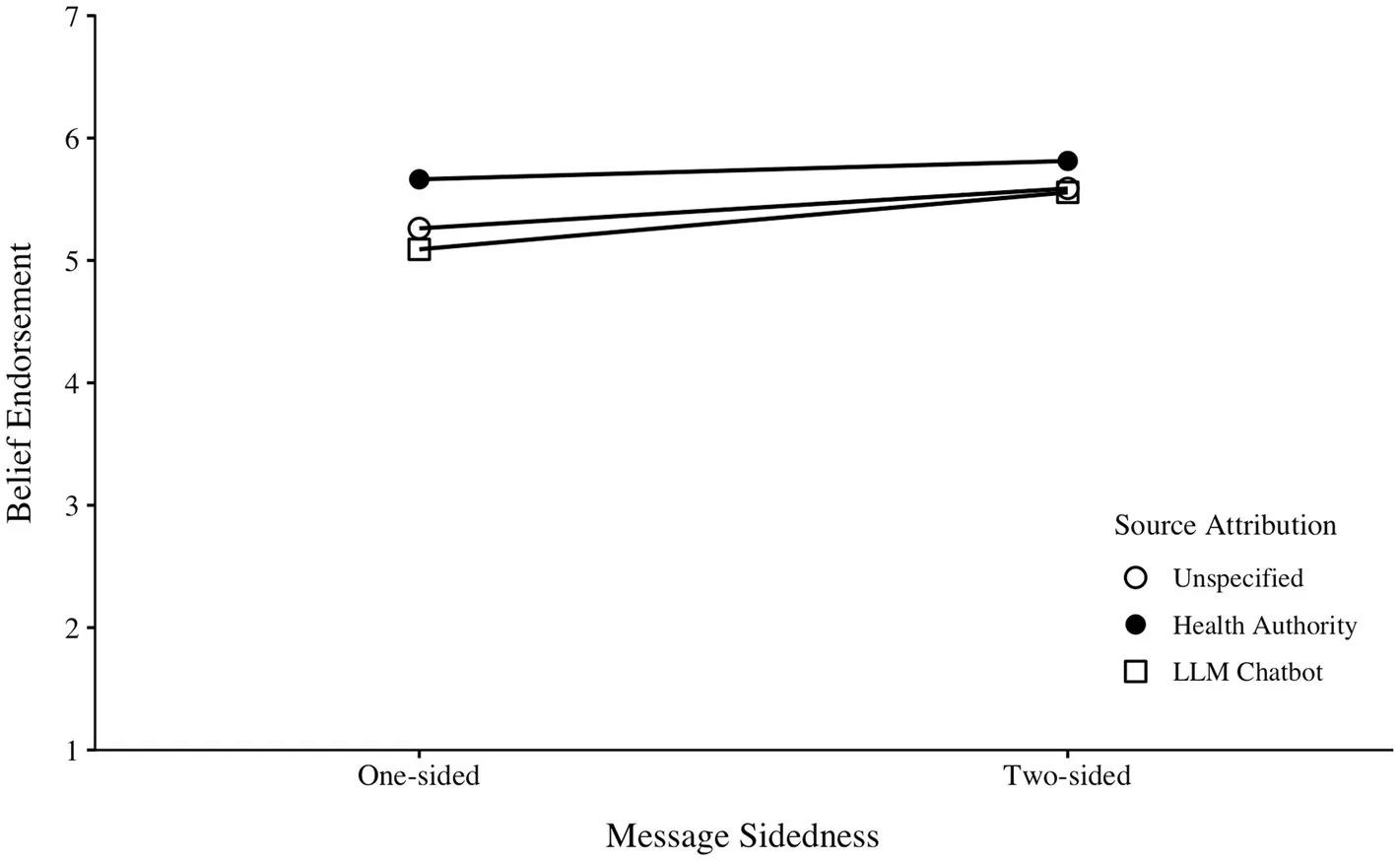
Effects of message sidedness and source attribution on belief endorsement.

The interaction between message sidedness and source attribution was not statistically significant, *F*(2, 537) = 0.764, *p* = 0.466, η^2^ = 0.003. The very small effect size suggests that the difference between one-sided and two-sided corrections did not meaningfully vary across source-attribution conditions. As shown in Figure 1, two-sided corrections generally yielded slightly higher post-exposure endorsement than one-sided corrections across the three source-attribution conditions, but the magnitude of this difference was not substantially different across LLM chatbot, health authority, and unspecified-source attributions.

### 3.2 Testing the psychological mechanisms

To further examine the psychological mechanisms underlying the observed main effects, two mediation analyses were conducted using Hayes’ PROCESS Model 4 with 5,000 bootstrap samples. The first mediation analysis examined whether perceived persuasiveness statistically mediated the association between message sidedness and post-exposure endorsement of the corrected claim. Results indicated a significant indirect effect through perceived persuasiveness, *b* = 0.278, 95% CI [0.131, 0.448], *p* < 0.001. This finding is consistent with the proposed message-structure pathway, suggesting that two-sided corrections were associated with higher perceived persuasiveness, which in turn was associated with higher post-exposure endorsement.

The second mediation analysis examined whether perceived source credibility statistically mediated the association between source attribution and post-exposure endorsement. Source attribution was dummy coded with unspecified source attribution as the reference category. The indirect effect through perceived source credibility was significant for health authority attribution, *b* = 0.757, 95% CI [0.579, 0.937], *p* < 0.001, and for LLM chatbot attribution, *b* = 0.208, 95% CI [0.040, 0.380], *p* = 0.014. These findings are consistent with the proposed source-attribution pathway, suggesting that source attribution was associated with endorsement partly through credibility-related evaluations of the attributed source (see Table 4).

**Table 4:** Total and indirect effects of message sidedness and source attribution on belief endorsement.

Effect	*b*	*SE*	*p*	95%*CI*	
				Lower	Upper
Total effects					
Message Sidedness → Belief Endorsement	**0.319**	0.105	**0.002**	0.125	0.536
Source: LLM Chatbot → Belief Endorsement	−0.101	0.128	0.433	−0.368	0.162
Source: Health Authority → Belief Endorsement	**0.307**	0.129	**0.018**	0.063	0.549
Indirect effects					
Message Sidedness →Perceived Persuasiveness → Belief Endorsement	**0.278**	0.079	**< 0.001**	0.131	0.448
Source: LLM Chatbot → Perceived Source Credibility → Belief Endorsement	**0.208**	0.085	**0.014**	0.040	0.380
Source: Health Authority → Perceived Source Credibility → Belief Endorsement	**0.757**	0.093	**< 0.001**	0.579	0.937

Source attribution was dummy coded with unspecified source as the reference category. Bootstrap confidence intervals were estimated based on 5,000 resamples. To reduce the risk of Type I error due to multiple mediation analyses, a Bonferroni-adjusted significance threshold (*α* = 0.025) was applied. Statistically significant coefficients are shown in bold.

## 4 Discussion

This study examined how corrective message structure and source attribution shape the acceptance of health misinformation correction in an AI-mediated communication context. The findings showed statistically significant but modest main effect of both message sidedness and source attribution. Refutational two-sided corrections produced higher endorsement than one-sided corrections, and corrections attributed to a government health authority produced higher endorsement than those attributed to an LLM chatbot or an unspecified source. However, the interaction between message sidedness and source attribution was not statistically significant, indicating that the advantage of two-sided correction did not meaningfully vary across attribution conditions.

These findings refine existing corrective persuasion research in two ways. First, they suggest that the persuasive benefit of refutational two-sided structure extends to an AI-attributed correction context, but the magnitude of this effect was small. Second, they qualify source-credibility models by showing that LLM chatbot attribution did not function equivalently to institutional health authority attribution. The study therefore does not suggest that AI fundamentally changes corrective persuasion. Rather, it shows that established message-structure and source-credibility processes continue to matter, while AI attribution introduces an important boundary condition for source-based persuasion. Specifically, message-structure mechanisms may generalize across source attributions, whereas source-credibility mechanisms are more sensitive to whether the source is institutional, algorithmic, or unspecified. This distinction contributes a more differentiated account of corrective persuasion in the AI era: AI attribution qualifies source-based credibility assumptions, but it does not appear to alter the basic persuasive function of refutational message structure.

### 4.1 LLM chatbot attribution as an algorithmic source cue

The source-attribution findings can be interpreted in relation to the Heuristic-Systematic Model (HSM) of information processing (Chaiken, 1980) and broader research on credibility heuristics. When individuals face high-stakes health decisions with limited time or cognitive resources, they rely on source heuristics, the mental shortcuts based on the perceived status and accountability of the speaker. Government agencies may also benefit from a legitimacy heuristic, as they are perceived as possessing not only expertise but also legal and moral accountability (Eisend, 2006). This reliance is especially relevant in health misinformation contexts, audiences often rely on source cues when they lack the expertise to independently evaluate technical or medical claims (Metzger et al., 2010; Sundar, 2008). In the present study, health authority attribution produced higher post-exposure endorsement than LLM chatbot attribution and unspecified attribution. This pattern is consistent with research showing that institutional health sources can benefit from perceived expertise, public responsibility, and accountability in health-risk communication (Holroyd et al., 2020).

LLM chatbot attribution occupied a more ambiguous position. Although AI systems may signal technical capacity, speed, and informational efficiency, these cues did not translate into the same level of post-exposure endorsement as health authority attribution. This pattern aligns with work on algorithmic trust and medical AI suggesting that people may hesitate to rely on automated systems in health contexts, particularly when accountability, responsibility, or human judgment is salient (Dietvorst et al., 2015; Longoni et al., 2019). The present findings therefore do not imply that AI-attributed health corrections are rejected outright. Rather, they suggest that AI attribution carries a different credibility profile: it may invite perceptions of technical competence, but it does not automatically supply the institutional legitimacy that audiences may associate with public health authorities.

The result of mediation models also consistent with this interpretation, although they should be read cautiously. Perceived source credibility statistically mediated the association between source attribution and endorsement, indicating that credibility-related evaluations were relevant to how participants responded to different source labels. However, LLM chatbot attribution did not produce a significant total endorsement advantage over unspecified attribution. This means that the significant indirect effect for AI attribution should not be interpreted as evidence that AI was more persuasive overall. A more cautious interpretation is that AI attribution activated some credibility-related inferences, but these were not strong enough to produce an overall advantage in endorsement.

### 4.2 Message sidedness and perceived persuasiveness

Beyond source attribution, the findings also highlight the role of message framing. We found that two-sided messages, which those that acknowledge a counterargument before refuting it, produce higher belief endorsement than one-sided factual corrections across source-attribution conditions. Classic and contemporary research suggests that messages acknowledging an opposing position and then rebutting it can appear more balanced, less biased, and more carefully reasoned than one-sided appeals (Allen, 1991; O’Keefe, 1999; Wallace et al., 2024). In the present study, two-sided corrections produced higher post-exposure endorsement than one-sided corrections, which suggests that this message structure may also be useful in health misinformation correction. At the same time, the size of this effect should not be overstated. The difference between one-sided and two-sided corrections was statistically significant but small. This matters theoretically because it suggests that refutational structure can improve correction acceptance, but it is unlikely to function as a decisive solution on its own. In contested health contexts, acknowledging and rebutting a misconception may help make a correction appear more persuasive, yet such effects remain bounded by other factors, including source credibility, prior beliefs, and the perceived relevance of the issue.

The indirect-effect further align with the proposed message-structure pathway. Two-sided corrections were associated with greater perceived persuasiveness, which in turn was associated with higher endorsement of the corrected claim. This pattern supports the proposed message-structure pathway, but it should not be read as definitive causal evidence. This structure likely reduces psychological reactance (Brehm, 1966) by making the user feel less like they are being coerced into a viewpoint and more like they are being presented with a balanced weighing of facts. Since AI cannot rely on a pre-existing legitimacy heuristic to persuade users, it could rely on the strength of its argumentation. In environments characterized by skepticism such as vaccine hesitancy, the rhetorical sophistication of the message becomes one of the important factor in belief revision. This suggests that for AI to be effective, it cannot merely retrieve facts. It should emulate the rhetorical complexity of human argumentation to effectively earn the trust.

### 4.3 Integrating source and structure in the AI era

The nonsignificant interaction between message sidedness and source attribution is useful for clarifying the relationship between source-based and message-based persuasion. Prior work suggests that message features and source cues can jointly shape how people evaluate information, particularly when recipients must judge both the credibility of the communicator and the quality of the argument (Flanagin et al., 2020). Research on AI source disclosure similarly shows that labeling a message as AI-generated or AI-assisted can alter credibility judgments, even when the message content itself is held constant (Lim and Schmälzle, 2024). Against this background, one plausible expectation was that refutational two-sided structure would be especially valuable for AI-attributed corrections, because AI sources may lack the institutional accountability associated with public health authorities.

The observed pattern did not support this stronger compensatory interpretation. Two-sided corrections tended to produce higher endorsement across attribution conditions, but this advantage did not meaningfully differ by whether the correction was attributed to a LLM chatbot, a health authority, or an unspecified source. This finding suggests a point of divergence from a strong source-message compensation account: improving the rhetorical structure of a correction may enhance perceived persuasiveness, but it does not appear to substitute for the credibility conferred by institutional source attribution. In other words, message design and source attribution seem to operate as complementary rather than interchangeable components of corrective communication.

This distinction helps position the present study within both two-sided persuasion research and AI-mediated health communication. Consistent with recent work on two-sided messages in misinformation correction, refutational structure appears to be useful beyond traditional persuasion contexts. However, the present findings also show that this usefulness has limits: two-sided structure did not uniquely strengthen AI-attributed corrections or eliminate the endorsement advantage of health authority attribution. For AI-assisted health communication, the implication is therefore not that sophisticated message design can overcome algorithmic credibility concerns, but that effective correction likely requires both credible attribution and persuasive message construction.

### 4.4 Practical implications

These findings offer practical implications for the deployment and design of AI-assisted health misinformation correction. First, given that LLM chatbot attribution produced lower endorsement than health authority attribution, developers and public health practitioners should avoid positioning chatbots as standalone experts in high-stakes health contexts. Instead, AI systems may function more appropriately as credibility bridges. Because the credibility gap appears to be partly rooted in human accountability and institutional legitimacy, AI interfaces should be designed to explicitly anchor corrections in established health authorities, professional guidelines, or official public health sources. For health authorities, this means that LLM chatbots may be useful as accessible communication interfaces for explaining and disseminating official guidance, rather than as replacements for institutional expertise. For platform designers, this also suggests the need for visible source labels, links to official guidance, and interface cues that distinguish AI-generated explanation from institutionally grounded evidence.

Second, the modest advantage of two-sided messaging indicates that accuracy alone may be insufficient for persuasion in contested health misinformation contexts. Message structure should therefore be considered alongside factual retrieval. Current AI-generated health responses often prioritize conciseness and directness, which may be useful for simple information seeking but less effective when users have already encountered misleading claims. Our findings suggest that health-oriented AI systems should be designed to adopt a refutational structure when correcting misinformation: briefly acknowledging the user’s concern or the common misconception, explaining why it may appear plausible, and then rebutting it with evidence-based information. For AI developers, this implies that correction-oriented systems should not merely retrieve accurate facts, but should also organize those facts in a way that addresses the misconception directly and makes the corrective argument appear balanced, transparent, and persuasive.

Finally, the findings point toward the potential value of adaptive message calibration, although such applications should be tested empirically in future research. Since refutational two-sided messages may be particularly relevant when users are skeptical or have already encountered competing claims, advanced AI systems could be designed to adjust the depth and structure of corrective responses based on the user’s expressed uncertainty, concern, or resistance. For example, when users ask about a contested health claim or express doubt toward official guidance, the system might provide a more elaborated refutational response that acknowledges the concern before correcting it. Conversely, for neutral information-seeking queries, a simpler factual response may be sufficient. Importantly, however, the nonsignificant interaction in the present study cautions against assuming that message structure alone can compensate for the lower credibility of AI attribution relative to health authority attribution. Effective AI-assisted correction is therefore likely to require both credible source attribution and carefully structured corrective messages.

### 4.5 Limitations

Despite its contributions, this study should be interpreted in light of several limitations that point toward important directions for future research. First, the experiment focused on a single health misinformation topic: whether influenza vaccination is safe during pregnancy. Although this issue represents a consequential and widely documented public health concern, responses to corrective messages may vary across health domains that differ in perceived risk, familiarity, or moralization (e.g., childhood vaccination, cancer treatment, or emerging infectious diseases). Future research should therefore examine whether the persuasive advantage of two-sided corrections generalizes across diverse misinformation contexts.

Second, the experimental design did not include a misinformation-onlyor no-exposure control condition, nor did it measure participants’ pre-existing beliefs about influenza vaccination during pregnancy before exposure. As a result, the present study cannot determine whether the corrective messages increased belief in the corrected claim, reduced misinformation beliefs, or maintained pre-existing beliefs. The findings should therefore be interpreted as evidence about the relative effectiveness of different correction designs and source attributions, rather than as evidence that correction exposure was more effective than no correction. Future studies should include baseline belief measures and appropriate control conditions to assess belief change more directly.

Third, the study relied on immediate self-reported measures of post-exposure endorsement and message evaluation. Although single-item belief endorsement measures are commonly used in misinformation correction research when assessing belief in a specific factual claim, this approach may still be susceptible to demand characteristics and short-term evaluative responses. Moreover, immediate post-exposure responses do not indicate whether the observed effects persist over time or translate into subsequent behavioral outcomes. Future research should incorporate delayed follow-up measures, behavioral indicators, multi-item belief measures where appropriate, or pre-post belief assessments to examine whether correction effects persist over time and translate into more durable belief revision.

## 5 Conclusion

As AI systems increasingly become the first responders for health information, understanding the psychology of their reception is critical. Our study demonstrates that the integration of AI has not rewritten the fundamental laws of persuasion: credibility remains deeply rooted in human accountability. However, this limitation is not a dead end. By combining transparent sourcing with sophisticated, two-sided argumentation, we can design AI systems that effectively navigate the complexities of human trust. Ultimately, the future of digital health communication lies not in replacing human expertise with algorithms, but in synthesizing the speed of AI with the rhetorical and institutional authority that only humans can provide.

## Data Availability

The raw data supporting the conclusions of this article will be made available upon reasonable request. Requests for data access should be directed to the corresponding author.

## References

[ref1] Allen M. (1991). Meta-analysis comparing the persuasiveness of one-sided and two-sided messages. West. J. Speech Commun. 55, 390–404. doi: 10.1080/10570319109374395

[ref3] Bode L. Vraga E. K. (2018). See something, say something: correction of global health misinformation on social media. Health Commun. 33, 1131–1140. doi: 10.1080/10410236.2017.1331312 28622038

[ref4] Brehm J. W. (1966). A Theory of Psychological Reactance. New York, NY: Academic Press.

[ref5] Cacciatore M. A. (2021). Misinformation and public opinion of science and health: approaches, findings, and future directions. Proc. Natl. Acad. Sci. 118:e1912437117. doi: 10.1073/pnas.1912437117, 33837143 PMC8053916

[ref7] Chaiken S. (1980). Heuristic versus systematic information processing and the use of source versus message cues in persuasion. J. Pers. Soc. Psychol. 39, 752–766. doi: 10.1037/0022-3514.39.5.752

[ref8] Chou W. Y. S. Oh A. Klein W. M. (2018). Addressing health-related misinformation on social media. JAMA 320, 2417–2418. doi: 10.1001/jama.2018.16865, 30428002

[ref9] Crowley A. E. Hoyer W. D. (1994). An integrative framework for understanding two-sided persuasion. J. Consum. Res. 20, 561–574. doi: 10.1086/209370

[ref10] Dietvorst B. J. Simmons J. P. Massey C. (2015). Algorithm aversion: people erroneously avoid algorithms after seeing them err. J. Exp. Psychol. Gen. 144, 114–126. doi: 10.1037/xge0000033, 25401381

[ref11] Ecker U. K. H. Lewandowsky S. Cook J. Schmid P. Fazio L. K. Brashier N. . (2022). The psychological drivers of misinformation belief and its resistance to correction. Nat. Rev. Psychol. 1, 13–29. doi: 10.1038/s44159-021-00006-y

[ref12] Ecker U. K. O'Reilly Z. Reid J. S. Chang E. P. (2020). The effectiveness of short-format refutational fact-checks. Br. J. Psychol. 111, 36–54. doi: 10.1111/bjop.12383, 30825195 PMC7004143

[ref13] Eisend M. (2006). Source credibility dimensions in marketing communication–a generalized solution. J. Empir. Gen. Mark. Sci. 10, 1–33.

[ref9001] Flanagin A. J. Winter S. Metzger M. J. (2020). Making sense of credibility in complex information environments: the role of message sidedness, information source, and thinking styles in credibility evaluation online. Information, Communication & Society, 23, 1038–1056. doi: 10.1080/1369118X.2018.1547411

[ref9002] Friestad M. Wright P. (1994). The persuasion knowledge model: How people cope with persuasion attempts. Journal of Consumer Research 21, 1–31. doi: 10.1086/209380

[ref14] Glass G. V. Peckham P. D. Sanders J. R. (1972). Consequences of failure to meet assumptions underlying the fixed effects analyses of variance and covariance. Rev. Educ. Res. 42, 237–288. doi: 10.3102/00346543042003237

[ref17] Holroyd T. A. Oloko O. K. Salmon D. A. Omer S. B. Limaye R. J. (2020). Communicating recommendations in public health emergencies: the role of public health authorities. Health Secur. 18, 21–28. doi: 10.1089/hs.2019.0073, 32078416 PMC7047090

[ref18] Hovland C. I. Lumsdaine A. A. Sheffield F. D. (1949). Experiments on Mass Communication. Princeton, NJ: Princeton University Press.

[ref20] Lewandowsky S. Ecker U. K. H. Seifert C. M. Schwarz N. Cook J. (2012). Misinformation and its correction: continued influence and successful debiasing. Psychol. Sci. Public Interest 13, 106–131. doi: 10.1177/1529100612451018, 26173286

[ref21] Lim S. Schmälzle R. (2024). The effect of source disclosure on evaluation of AI-generated messages. Comput. Hum. Behav. Artif. Hum. 2:100058. doi: 10.1016/j.chbah.2024.100058

[ref22] Longoni C. Bonezzi A. Morewedge C. K. (2019). Resistance to medical artificial intelligence. J. Consum. Res. 46, 629–650. doi: 10.1093/jcr/ucz013

[ref23] Metzger M. J. Flanagin A. J. Medders R. B. (2010). Social and heuristic approaches to credibility evaluation online. J. Commun. 60, 413–439. doi: 10.1111/j.1460-2466.2010.01488.x

[ref24] Nass C. Moon Y. (2000). Machines and mindlessness: social responses to computers. J. Soc. Issues 56, 81–103. doi: 10.1111/0022-4537.00153

[ref25] O’Keefe D. J. (1999). How to handle opposing arguments in persuasive messages: a meta-analytic review of the effects of one-sided and two-sided messages. Ann. Int. Commun. Assoc. 22, 209–249. doi: 10.1080/23808985.1999.11678963

[ref27] Sundar S. S. (2008). “The MAIN model: a heuristic approach to understanding technology effects on credibility,” in Digital media, Youth, and Credibility, eds. Metzger M. Flanagin A. (Cambridge, MA: MIT Press), 73–100.

[ref28] Sussman S. W. Siegal W. S. (2003). Informational influence in organizations: an integrated approach to knowledge adoption. Inf. Syst. Res. 14, 47–65. doi: 10.1287/isre.14.1.47.14767, 19642375

[ref29] Tam T. Y. C. Sivarajkumar S. Kapoor S. Stolyar A. V. Polanska K. McCarthy K. R. . (2024). A framework for human evaluation of large language models in healthcare derived from literature review. NPJ Digit. Med. 7:258. doi: 10.1038/s41746-024-01258-7 39333376 PMC11437138

[ref30] Tang R. Fang Y. Sun J. Bode L. Vraga E. K. (2025). Automating accuracy: scalable approaches to correcting disinformation with artificial intelligence on social media. J. Mass Commun. Q. 102, 1044–1070. doi: 10.1177/10776990251359660

[ref31] Vraga E. K. Bode L. (2017). Using expert sources to correct health misinformation in social media. Sci. Commun. 39, 621–645. doi: 10.1177/1075547017731776

[ref32] Vraga E. K. Bode L. (2020). Correction as a solution for health misinformation on social media. Am. J. Public Health 110, S278–S280. doi: 10.2105/AJPH.2020.305916, 33001724 PMC7532323

[ref33] Wallace L. E. Hinsenkamp L. Wegener D. T. Braun Z. (2024). Effects of message-sidedness on perceived source bias: when presenting two sides does versus does not alleviate concerns about bias. Personal. Soc. Psychol. Bull. 50, 807–820. doi: 10.1177/01461672231155389, 36803257

[ref34] Walter N. Brooks J. J. Saucier C. J. Suresh S. (2020). Evaluating the impact of attempts to correct health misinformation on social media: a meta-analysis. Health Commun. 36, 1776–1784. doi: 10.1080/10410236.2020.1794553 32762260

[ref35] Wang W. Huang Y. (2021). Countering the “harmless e-cigarette” myth: the interplay of message format, message sidedness, and prior experience with e-cigarette use in misinformation correction. Sci. Commun. 43, 170–198. doi: 10.1177/1075547020974384

[ref36] Wang Y. Rodríguez de Gil P. Chen Y. H. Kromrey J. D. Kim E. S. Pham T. . (2017). Comparing the performance of approaches for testing the homogeneity of variance assumption in one-factor ANOVA models. Educ. Psychol. Meas. 77, 305–329. doi: 10.1177/0013164416645162, 29795915 PMC5965542

[ref9003] Xiao X. Su Y. (2021). Integrating Reasoned Action Approach and Message Sidedness in the Era of Misinformation: The Case of HPV Vaccination Promotion. Journal of Health Communication, 26, 371–380. doi: 10.1080/10810730.2021.1950873 34252003

[ref39] Xu M. Petty R. E. (2024). Two-sided messages promote openness for a variety of deeply entrenched attitudes. Personal. Soc. Psychol. Bull. 50, 215–231. doi: 10.1177/01461672221128113, 36214520

[ref40] Xu M. Petty R. E. (2025). Using two-sided messages to facilitate misinformation correction for strongly held beliefs. J. Exp. Soc. Psychol. 121:104807. doi: 10.1016/j.jesp.2025.104807

[ref41] Yun H. S. Bickmore T. (2025). Online health information–seeking in the era of large language models: cross-sectional web-based survey study. J. Med. Internet Res. 27:e68560. doi: 10.2196/68560, 40163112 PMC11997521

[ref42] Zhang K. Z. Zhao S. J. Cheung C. M. Lee M. K. (2014). Examining the influence of online reviews on consumers’ decision-making: a heuristic–systematic model. Decis. Support. Syst. 67, 78–89. doi: 10.1016/j.dss.2014.08.005

